# Editorial: Critical assessment of massive data analysis (CAMDA) annual conference 2021

**DOI:** 10.3389/fgene.2023.1154398

**Published:** 2023-02-16

**Authors:** Paweł P. Łabaj, Joaquin Dopazo, Wenzhong Xiao, David P. Kreil

**Affiliations:** ^1^ Małopolska Centre of Biotechnology, Jagiellonian University, Kraków, Lesser Poland, Poland; ^2^ Computational Medicine Platform, Andalusian Public Foundation Progress and Health-FPS, Sevilla, Spain; ^3^ Institute of Biomedicine of Seville, IBiS, University Hospital Virgen del Rocío/CSIC/University of Sevilla, Sevilla, Spain; ^4^ Genome Technology Center, School of Medicine, Stanford University, Palo Alto, CA, United States; ^5^ Massachusetts General Hospital, Harvard Medical School, Boston, MA, United States; ^6^ Department of Biotechnology, Boku University Vienna, Vienna, Austria

**Keywords:** data analysis, benchmarking, open-ended competition, disease mechanisms, COVID-19, drug induced liver injury (DILI), machine learning, artificial intelligence—AI

In its unique approach through open-ended data analysis contests, CAMDA in 2021 again highlighted key challenges in analysing massive data in the life sciences. Since its inaugural meeting at Duke University in 2000, CAMDA has become a major fixture (Johnson and Lin, 2001; Tilstone, 2003; Editorial feature, 2008; Mangul et al., 2019) and runs yearly. Celebrating its 20th conference meeting at ISMB 2021, it has become the longest series of international data analysis competitions, featuring more challenges over time than even the well-known biannual CASP (Moult et al., 1995). This Research Topic collects selected highlights from the conference as original research papers.

The increasing relevance of Big Data in the modern life sciences keeps CAMDA topical, addressing one of the grand challenges in the field. Improvements in both the productivity and accessibility of genome-scale assays together with the recognition that high-dimensional profiling requires large sample sizes are driving ever increasing data set sizes. Still, the data analysis bottleneck limits the rate with which new medical and biological insights can be found. CAMDA tackles this challenge head on, introducing and evaluating new approaches and solutions. Specifically, the conference presents new techniques in bioinformatics, data analysis, and statistics for large data sets, combining multiple data sources, and effective computational inference. Every year, unique data sets are compiled and selected, with multiple academic research groups analysing the same data competitively. The most insightful analyses are then selected by vote amongst the 100–200 researchers attending the CAMDA conference sessions.

One challenge this year sought insights on the molecular response to COVID-19. Delegates explored and combined existing mechanistic maps and large-scale expression profiles. We here present a highlight from that challenge, where Dongyuan Wu et al. focus on the response of T cells to SARS-CoV-2. The authors detected pairwise differentially expressed genes by single-cell RNA-seq of CD4^+^ and CD8^+^ T cells, comparing a healthy control group to patients with either mild or severe COVID. A deep learning algorithm identified patterns of differentially expressed genes distinguishing the different disease states. The emerging co-expression networks for CD4^+^ T cells yielded 6 modules for healthy patients, 4 for mild disease, and 1 module for severe disease. For CD8^+^ T cells, they found 6, 4, and 3 modules. An identification of hub genes implicated TNF, CCL4, XCL1, and IFITM1 in the SARS-CoV-2 response. IFITM1 was already known to inhibit infection by enveloped viruses, including other coronaviruses. The newly identified networks thus yielded novel COVID related gene clusters and patterns of gene connectivity for further investigation. Further development of machine learning exploiting disease maps, molecular profiles, and patient data will continue to advance our understanding of disease mechanisms and support drug repurposing.

In the development of novel drugs, however, unexpected Drug-induced liver injury (DILI) remains a major disruptor. In collaboration with the US-FDA, CAMDA has approached this topic from a range of complementary angles in recent years. DILI is an important class of adverse drug reactions. In fact, it is the most frequent cause of acute liver failure in many Western countries. Manual trawling of the literature is the main route of deriving information on DILI from research studies, yet this is an inefficient process that scales badly and is prone to human error. Therefore, hope has been placed in automated assistance by AI models identifying articles of likely relevance to DILI amongst the many million research articles published. We here report advances from the related CAMDA 2021 challenge. Training data were compiled with the help of the US-FDA NCTR and support by IARAI (www.fda.gov/about-fda/office-chief-scientist/national-center-toxicological-research, www.iarai.ac.at).

Sanjay Rathee et al. apply customized token generation for word sets of varying lengths after standard NLP segmentation, lemmatization, and filtering. Superset patterns are identified from these word sets by a distributed *a priori* algorithm, with higher weights for the curated sources. Scores are then considered by an ensemble of classifiers in nested cross-validation. In another contribution by this group, Nicholas Katritsis et al. start by extracting mentions of chemical and disease terms *via* PubTator, and embed their TF-IDF representations with node2vec in the context of corpora derived from Chemical Checker, MeSH, and the Comparative Toxicogenomics Database. The neural network classifier exploiting these chemical and disease embeddings outperformed one based on text alone.

Malik Yousef et al., in contrast, replace the traditional Bag-of-words approach with a Bag-of-topics. Topics are extracted by LDA, then topic-specific data sub-sets are generated and, based on these, topics are scored and ranked by Random Forest with Monte-Carlo stratified cross-validation. Finally, the best ranked topics are used in a bag-of-topics Random Forest classifier, outperforming comparable bag-of-word classifiers.

All three papers achieved F1 scores in the range of 88%–91% on independent validation sets. Further tests with more imbalanced class distributions will facilitate real-world applications.

An essential part of CAMDA, however, is its open-ended data analysis of complex data sets, often featuring novel technological platforms, exceptionally large cohorts, and heterogeneous data sources and types. Both contestants and other interested researchers are welcome at the meeting. In this context of fruitful debate, it is precisely its open-ended character that allows CAMDA to foster disruptive and innovative ideas, inspiring discussions and analysis ideas that go beyond the initial premises. Consequently, the winning CAMDA contributions are not already determined by algorithm performance but consider novelty and insights presented. This winner of CAMDA 2021 identified exciting new therapeutic leads for COVID by integrating the mechanistic disease map with the viral-human metabolic network, and these leads are currently being validated pre-clinically before publication of a manuscript in the CAMDA Proceedings. We are proud of this year’s Research Topic of papers.
